# Development of a predictive model for systemic lupus erythematosus incidence risk based on environmental exposure factors

**DOI:** 10.1136/lupus-2024-001311

**Published:** 2024-11-20

**Authors:** Ying Zhang, Cheng Zhao, Yu Lei, Qilin Li, Hui Jin, Qianjin Lu

**Affiliations:** 1 Department of Epidemiology and Biostatistics, Nanjing Medical University, Nanjing, China; 2 Hospital for Skin Diseases, Institute of Dermatology, Chinese Academy of Medical Sciences and Peking Union Medical College, Nanjing, China; 3 Key Laboratory of Basic and Translational Research on Immune-Mediated Skin Diseases, Chinese Academy of Medical Sciences, Nanjing, China; 4 Jiangsu Key Laboratory of Molecular Biology for Skin Diseases and STIs, Nanjing, China; 5 Hunan Key Laboratory of Medical Epigenomics, Department of Dermatology, The Second Xiangya Hospital of Central South University, Changsha, China

**Keywords:** Lupus Erythematosus, Systemic, Risk Factors, Epidemiology

## Abstract

**Objective:**

Systemic lupus erythematosus (SLE) is an autoimmune disease characterised by a loss of immune tolerance, affecting multiple organs and significantly impairing patients’ health and quality of life. While hereditary elements are essential in the onset of SLE, external environmental influences are also significant. Currently, there are few predictive models for SLE that takes into account the impact of occupational and living environmental exposures. Therefore, we collected basic information, occupational background and living environmental exposure data from patients with SLE to construct a predictive model that facilitates easier intervention.

**Methods:**

We conducted a study comparing 316 individuals diagnosed with SLE and 851 healthy volunteers in a case–control design, collecting their basic information, occupational exposure history and environmental exposure data. Subjects were randomly allocated into training and validation groups using a 70/30 split. Using three-feature selection methods, we constructed four predictive models with multivariate logistic regression. Model performance and clinical utility were evaluated via receiver operating characteristic, calibration and decision curves. Leave-one-out cross-validation further validated the models. The best model was used to create a dynamic nomogram, visually representing the predicted relative risk of SLE onset.

**Results:**

The ForestMDG model demonstrated strong predictive ability, with an area under the curve of 0.903 (95% CI 0.880 to 0.925) in the training set and 0.851 (95% CI 0.809 to 0.894) in the validation set, as indicated by model performance evaluation. Calibration and decision curves demonstrated accurate results along with practical clinical value. Leave-one-out cross-validation confirmed that the ForestMDG model had the best accuracy (0.8338). Finally, we developed a dynamic nomogram for practical use, which is accessible via the following link: https://yingzhang99321.shinyapps.io/dynnomapp/.

**Conclusion:**

We created a user-friendly dynamic nomogram for predicting the relative risk of SLE onset based on occupational and living environmental exposures.

**Trial registration number:**

ChiCTR2000038187.

**WHAT IS ALREADY KNOWN ON THIS TOPIC**

- Systemic lupus erythematosus (SLE) severely impacts patients’ health and quality of life. While genetic factors play a crucial role in its onset, exposure to environmental and occupational risk factors also significantly increases the risk of developing SLE. Existing predictive models often fail to adequately consider the influence of these factors on SLE development. Therefore, our study aims to develop and validate a predictive model that includes occupational and environmental exposure data, thereby filling this gap and providing an effective tool for early intervention and risk assessment.

**WHAT THIS STUDY ADDS**

- We identified a close relationship between occupational and environmental exposure factors and the onset of SLE. Based on these factors, we developed and compared four risk assessment predictive models. The best-performing model achieved an area under the curve of 0.903 in the training set and 0.851 in the validation set. Additionally, we created a dynamic nomogram for practical application, which can be accessed via the following link: https://yingzhang99321.shinyapps.io/dynnomapp/.

**HOW THIS STUDY MIGHT AFFECT RESEARCH, PRACTICE OR POLICY**

- This study highlights the significant influence of occupational and living environmental factors on the risk of developing SLE. The predictive model can help assess SLE relative risk under everyday environmental exposures, guiding high-risk individuals to modify their health habits. This serves as a primary prevention strategy to reduce the incidence of SLE.

## Introduction

 Systemic lupus erythematosus (SLE) is a multifaceted autoimmune disorder marked by a breakdown in immune tolerance, resulting in the generation of numerous autoantibodies and immune complexes within the body.^1 2^ These abnormal immune responses can impact one or more organs, causing inflammation and significantly jeopardising patients’ health and overall well-being.^3^ The clinical manifestations of SLE are highly heterogeneous, impacting various organ systems such as the skin, joints, central nervous system, vascular system and kidneys. The disease course may present as chronic progression or recurrent flare-ups and remissions, imposing significant physical and psychological burdens on patients.^4 5^ Although SLE is relatively rare, its impact on affected populations is significant. According to global estimates, SLE occurs at an annual rate of approximately 5.14 cases per 100 000 people annually, leading to about 400 000 new diagnoses each year. Meanwhile, the global prevalence of SLE is as high as 43.7 cases per 100 000 person-years, with an estimated total of 3.41 million patients with SLE worldwide.^6^ SLE leads to a sharp increase in personal medical expenses and causes a loss of labour capacity, significantly diminishing patients’ quality of life and markedly escalating the disease burden.^7^,^9^

The aetiology of SLE is extremely complex and multifaceted, involving genetics, hormones, lifestyle, living environmental exposures and gut microbiota, among other factors.^10^,^14^ While hereditary elements are known to be significant in the development of SLE, research data suggest that even among identical twins, the concordance rate for SLE is only 25%–30%, contrasting sharply with almost no concordance among fraternal twins.^3^ This finding suggests that although genetic factors are important, the occurrence and development of SLE are not entirely determined by genetics. Instead, external environmental exposure factors exert a greater influence on the development of SLE.

Studies examining the link between occupational exposure and SLE indicate that exposure to respirable crystalline silica correlates positively with autoimmune disease risk.^15^ The risk of SLE was found to be as high as 5.33-fold in those with high exposure to herbicides, and it increased to 2.97-fold in those with high exposure to pesticides.^16^ Additionally, exposure to organic solvents is highly linked to a greater likelihood of developing SLE.^17 18^ Beyond occupational exposure, factors such as smoking, exogenous oestrogen intake, irregular dietary habits and sleep abnormalities have been suggested as potential risk factors for SLE.^13 19^ Living air pollution is also not negligible in the development of lupus. A population-based cohort study conducted in Taiwan indicated that higher levels of particulate matter (PM), carbon monoxide and nitrogen dioxide all increased the risk of SLE.^20^ Our related studies have found that lifestyle-related polycyclic aromatic hydrocarbons’ (PAHs) exposure has a greater impact on SLE. The level of PAHs exposure gradually increases from healthy controls to patients with non-active SLE and active SLE.^21^

Currently, the prediction of SLE risk primarily focuses on genetic factors and basic lifestyle factors, such as body mass index, smoking, alcohol use, sleep patterns, socioeconomic status, oral contraceptive use and age at menarche.^22 23^ However, these models often overlook occupational exposure factors and living environmental factors. Our preliminary investigation revealed that both occupational and living environmental exposures exert a substantial influence on the onset and progression of SLE, and these factors are modifiable. Therefore, we are dedicated to using basic demographic information, occupational backgrounds, and living environmental exposure data from patients with SLE to develop a practical and easy-to-use predictive model for the relative risk of SLE onset. By comparing and evaluating the performance of different models, we aim to better understand how these exposure factors influence the risk of SLE occurrence, thereby providing scientific evidence for precise prevention and early intervention.

## Methods

### Population and study design

Due to the low prevalence of SLE, a case–control study design was employed to enhance the efficiency and statistical power of the study. We recruited patients with SLE from the dermatology, rheumatology and nephrology outpatient clinics and wards of the second Xiangya Hospital of Central South University, as well as from individuals undergoing health assessments at the hospital’s Health Management Center. Over 90% of the participants were residents of Hunan Province, China. To be included, patients with SLE had to be 18 years or older with a clinical diagnosis of SLE confirmed by a minimum of one attending physician or senior medical staff. Healthy control subjects also needed to be 18 years or older. Exclusion criteria for the control group included refusal to participate as well as the presence of neurological or psychiatric disorders, or physical disabilities that could interfere with the assessment of environmental exposures. Data collection was conducted from 30 November 2020 to 22 October 2022. It was additionally registered in the China Clinical Trial Registry under registration number ChiCTR2000038187. We ensured that all participants provided informed consent before their involvement.

### Data collection

We developed two electronic surveys to investigate occupational and environmental exposure factors in both the case and control groups. Participants can complete the questionnaires by scanning QR codes. Additionally, this method ensures that there will be no missing data. While the surveys are designed for self-completion, legal representatives may complete them and sign the informed consent form if participants are unable to do so due to illiteracy or other reasons. The survey included demographic information, detailed occupational exposure history and data on various living environment exposures. These environmental data covered air quality (such as outdoor air pollution at residential or workplace locations), indoor air quality (including ventilation conditions and kitchen exhaust systems) and residential factors, such as the presence of new furniture, cooking methods (eg, gas, electric or solid fuel) and heating sources (classified by type, such as wood-burning stoves, central heating or electric heaters). Most of the data collected were in categorical form, which facilitates the classification and analysis of participants’ exposure levels. Data-related content and definitions are found in online supplemental table S1 and our published article.^21^

### Statistical analysis

First, we performed statistical and univariate analyses on the baseline data. Continuous variables with a normal distribution are shown as mean±SD and analysed with the t test. Variables that do not follow a normal distribution are presented as median (IQR) and examined using the Mann-Whitney U test. Categorical variables, expressed as frequency (percentage, %), are compared using the χ^2^ test or Fisher’s exact test.

Variables with a p value less than 0.05 were selected based on the results of the multivariable analysis, in which age and gender were adjusted as covariates. Subjects were allocated at random into training and validation groups using a 70/30 split. We used forward and backward stepwise multivariate logistic regression to select variables, ranking them by the absolute value of their coefficients and selecting the top 10 for the Step model in the training set. Lasso regression was applied to further refine variables with p<0.05, again selecting the top 10 for the Lasso model. We used random forest to rank variable importance, selecting the top 10 variables from MeanDecreaseAccuracy and MeanDecreaseGini rankings for the ForestMDA and ForestMDG models, respectively.

**Figure 1:**
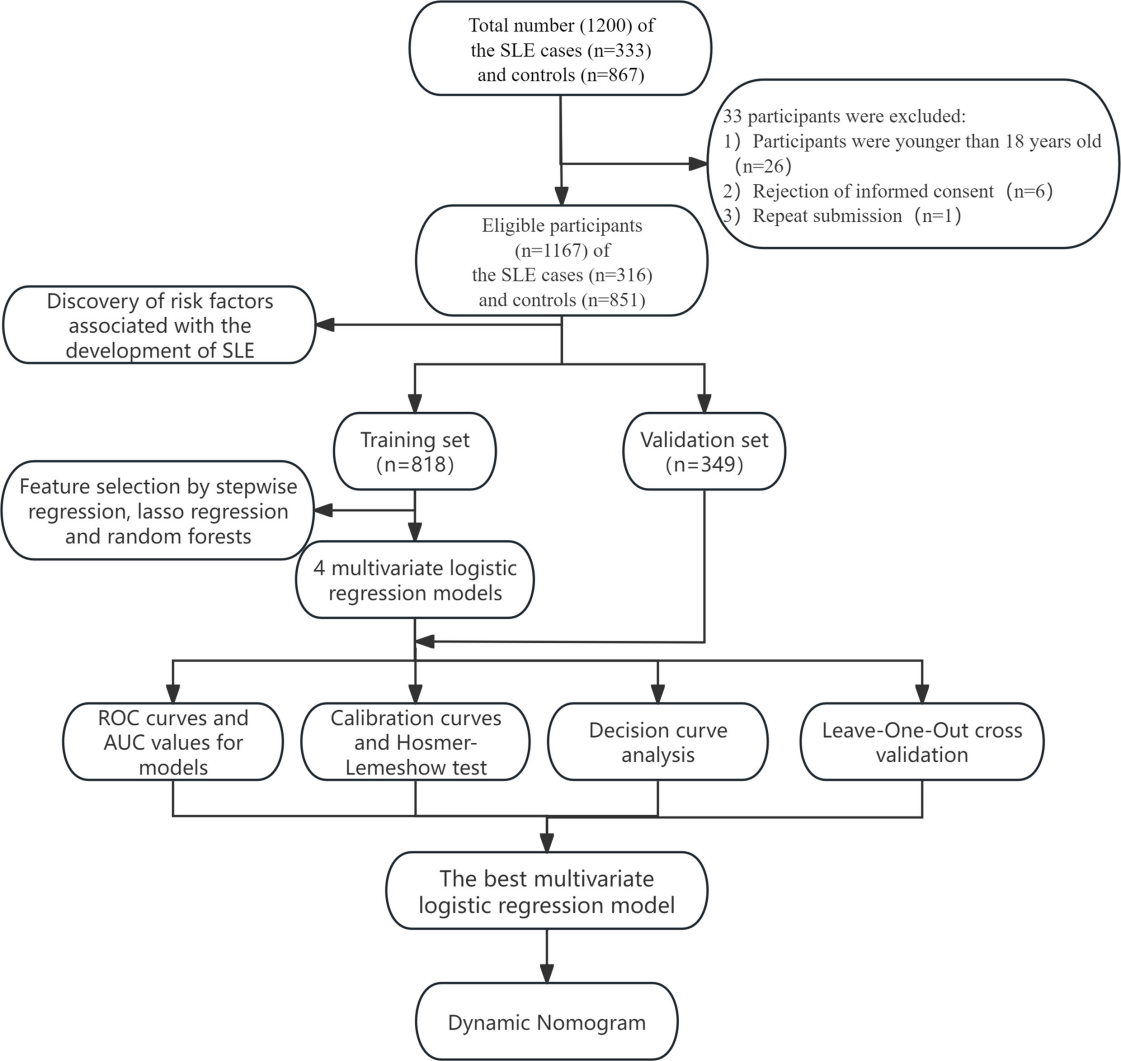
Flow diagram of the study. AUC, area under the ROC curve; ROC, receiver operating characteristic; SLE, systemic lupus erythematosus.

We plotted receiver operating characteristic curves and calibration curves for both sets and calculated the area under the curves (AUC) to gauge model effectiveness. The models’ accuracy and goodness-of-fit were assessed with calibration curves of actual versus predicted SLE probabilities, along with decision curve analysis (DCA) to appraise clinical effectiveness by calculating net benefit across threshold probabilities (figure 1).

Statistical analysis and plotting were conducted with R software (V.4.3.2), utilising packages such as Moonbook, MASS, glmnet, randomForest, ResourceSelection, pROC, rms, rmda, caret and ggplot2. Statistical significance was defined as p<0.05 (two-tailed).

## Results

### Patient characteristics

From individuals diagnosed with SLE, we obtained 333 questionnaires, and from the control group, we collected 867. During data processing, we excluded two blank surveys in the SLE cohort and four in the healthy cohort due to participants selecting ‘opted out of the study’ on the informed consent form as well as one duplicate survey in the SLE cohort. Additionally, we removed 14 questionnaires from the case group and 12 from the control group with participants under 18 years of age. Ultimately, we conducted an analysis on 316 questionnaires from patients with SLE and 851 questionnaires from the control group. After random allocation, the training set included 818 participants and the validation set included 349 participants. We then compared the consistency between the two datasets (online supplemental table S2–3).

**Figure 2:**
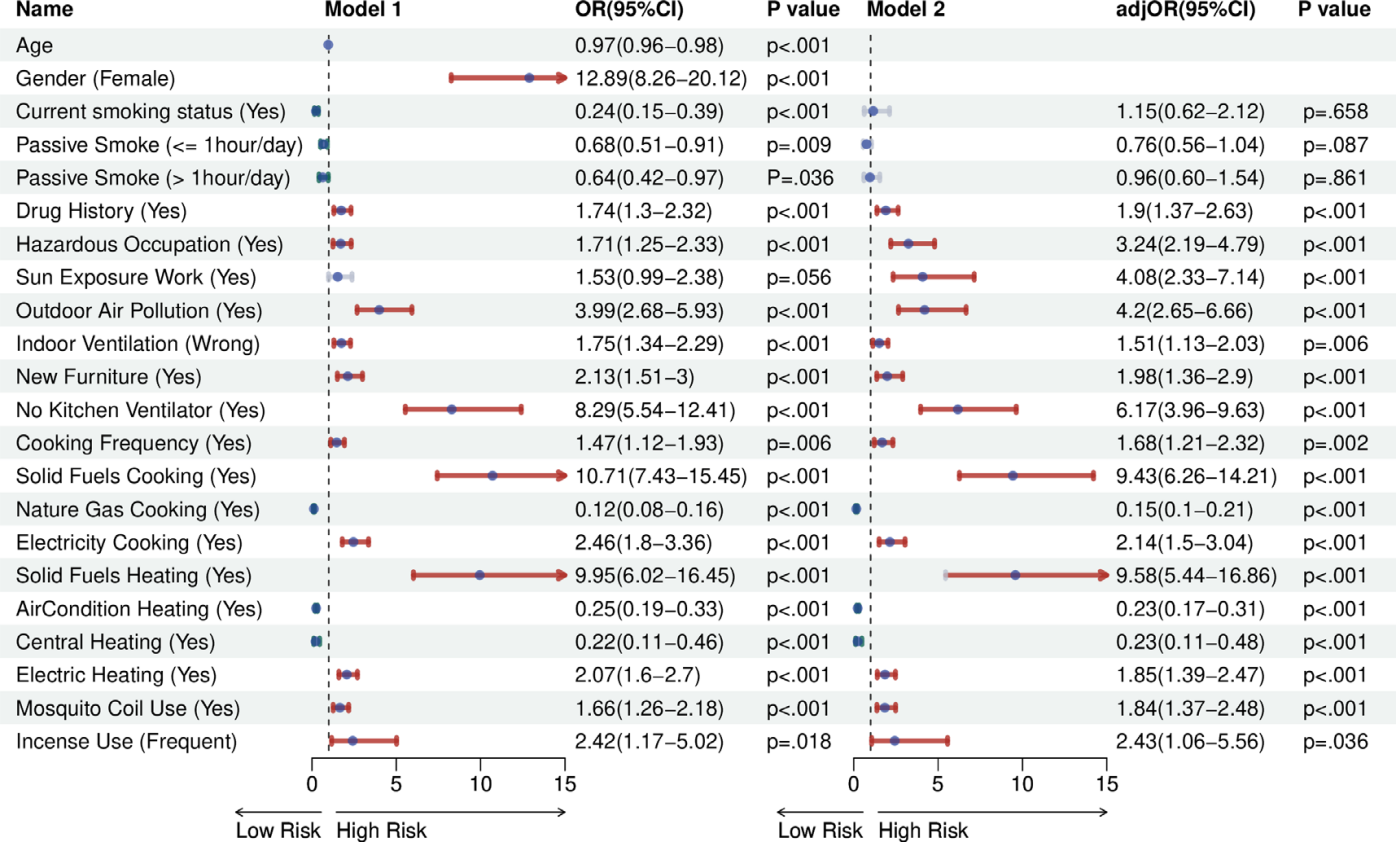
Predictors of the development of SLE in the entire dataset. Model 1: ORs were not adjusted for covariates. Model 2: ORs were adjusted for gender and age. SLE, systemic lupus erythematosus.

We collected some demographic information, occupational exposure data and daily environmental exposure factors. Table 1 presents the baseline characteristics of participants in both the training and validation sets. Among the 1167 participants, 61.35% were women. The overall cohort had a median age of 36 years (IQR: 30–45 years) and included 316 individuals diagnosed with SLE. Univariate analysis revealed significant correlation between SLE development and factors such as current smoking status, secondhand smoke exposure, drug history, occupational exposure to hazardous substances, outdoor air pollution, indoor air ventilation, new furniture, kitchen ventilation system, frequency of cooking, traditional solid fuel cooking, natural gas cooking, appliance cooking, traditional solid fuel heating, air conditioning heating, central heating, electric heating, mosquito coils and aroma use (online supplemental table S4). Further calculation of ORs in figure 2 showed that current smoking status and passive smoking were not significantly associated with SLE development after adjusting for age and gender. However, all other factors demonstrated significant associations with SLE development. After accounting for age and gender, significant contributors to SLE included drug history (OR: 1.9; 95% CI 1.37 to 2.63), occupational exposure to hazardous substances (OR: 3.24; 95% CI 2.19 to 4.79), sun-exposed occupations (OR: 4.08; 95% CI 2.33 to 7.14), outdoor air pollution (OR: 4.2; 95% CI 2.65 to 6.66), poor indoor air circulation (OR: 1.51; 95% CI 1.13 to 2.03), new furniture (OR: 1.98; 95% CI 1.36 to 2.9), absence of a kitchen ventilation system (OR: 6.17; 95% CI 3.96 to 9.63), cooking more than two times per week (OR: 1.68; 95% CI 1.21 to 2.32), traditional solid fuel cooking (OR: 9.43; 95% CI 6.26 to 14.21), electricity cooking (OR: 2.14; 95% CI 1.5 to 3.04), traditional solid fuel heating (OR: 9.58; 95% CI 5.44 to 16.86), electric heating (OR: 1.85; 95% CI 1.39 to 2.47), mosquito coil consumption (OR: 1.84; 95% CI 1.37 to 2.48), and frequent incense burning (OR: 2.43; 95% CI 1.06 to 5.56). Protective factors of SLE included natural gas cooking (OR: 0.15; 95% CI 0.1 to 0.21), air conditioning heating (OR: 0.23; 95% CI 0.17 to 0.31) as well as central heating (OR: 0.23; 95% CI 0.11 to 0.48).

**Table 1:** Baseline characteristics of the overall study population, divided according to the presence or absence of SLE disease

Group	Control	SLE	Total	P value
	(N=851)	(N=316)	(N=1167)	
Age	37.00 (31.00;47.00)	34.00 (26.00;42.00)	36.00 (30.00;45.00)	<0.001
Gender				<0.001
Male	428 (50.29%)	23 (7.28%)	451 (38.65%)	
Female	423 (49.71%)	293 (92.72%)	716 (61.35%)	
Current smoking status				<0.001
No	671 (78.85%)	297 (93.99%)	968 (82.95%)	
Yes	180 (21.15%)	19 (6.01%)	199 (17.05%)	
Passive smoke				0.011
No	414 (48.65%)	185 (58.54%)	599 (51.33%)	
≤1 hour/day	318 (37.37%)	97 (30.70%)	415 (35.56%)	
>1 hour/day	119 (13.98%)	34 (10.76%)	153 (13.11%)	
Drug history				<0.001
No	672 (78.97%)	216 (68.35%)	888 (76.09%)	
Yes	179 (21.03%)	100 (31.65%)	279 (23.91%)	
Hazardous occupation				0.001
No	710 (83.43%)	236 (74.68%)	946 (81.06%)	
Yes	141 (16.57%)	80 (25.32%)	221 (18.94%)	
Sun exposure work				0.072
No	789 (92.71%)	282 (89.24%)	1071 (91.77%)	
Yes	62 (7.29%)	34 (10.76%)	96 (8.23%)	
Outdoor air pollution				<0.001
No	801 (94.12%)	253 (80.06%)	1054 (90.32%)	
Yes	50 (5.88%)	63 (19.94%)	113 (9.68%)	
Indoor ventilation				<0.001
Good	599 (70.39%)	182 (57.59%)	781 (66.92%)	
Wrong	252 (29.61%)	134 (42.41%)	386 (33.08%)	
New furniture				<0.001
No	754 (88.60%)	248 (78.48%)	1002 (85.86%)	
Yes	97 (11.40%)	68 (21.52%)	165 (14.14%)	
No kitchen ventilator				<0.001
No	812 (95.42%)	226 (71.52%)	1038 (88.95%)	
Yes	39 (4.58%)	90 (28.48%)	129 (11.05%)	
Cooking frequency				0.007
≤2 days/week	608 (71.45%)	199 (62.97%)	807 (69.15%)	
>2 days/week	243 (28.55%)	117 (37.03%)	360 (30.85%)	
Solid fuels cooking				<0.001
No	802 (94.24%)	191 (60.44%)	993 (85.09%)	
Yes	49 (5.76%)	125 (39.56%)	174 (14.91%)	
Nature gas cooking				<0.001
No	71 (8.34%)	138 (43.67%)	209 (17.91%)	
Yes	780 (91.66%)	178 (56.33%)	958 (82.09%)	
Electricity cooking				<0.001
No	734 (86.25%)	227 (71.84%)	961 (82.35%)	
Yes	117 (13.75%)	89 (28.16%)	206 (17.65%)	
Solid fuels heating				<0.001
No	829 (97.41%)	250 (79.11%)	1079 (92.46%)	
Yes	22 (2.59%)	66 (20.89%)	88 (7.54%)	
Air condition heating				<0.001
No	302 (35.49%)	218 (68.99%)	520 (44.56%)	
Yes	549 (64.51%)	98 (31.01%)	647 (55.44%)	
Central heating				<0.001
No	762 (89.54%)	308 (97.47%)	1070 (91.69%)	
Yes	89 (10.46%)	8 (2.53%)	97 (8.31%)	
Electric heating				<0.001
No	535 (62.87%)	142 (44.94%)	677 (58.01%)	
Yes	316 (37.13%)	174 (55.06%)	490 (41.99%)	
Mosquito coil use				<0.001
No	373 (43.83%)	101 (31.96%)	474 (40.62%)	
Yes	478 (56.17%)	215 (68.04%)	693 (59.38%)	
Incense use				0.025
Infrequent	835 (98.12%)	302 (95.57%)	1137 (97.43%)	
Frequent	16 (1.88%)	14 (4.43%)	30 (2.57%)	

SLEsystemic lupus erythematosus

### Model variable screening and development

**Figure 3:**
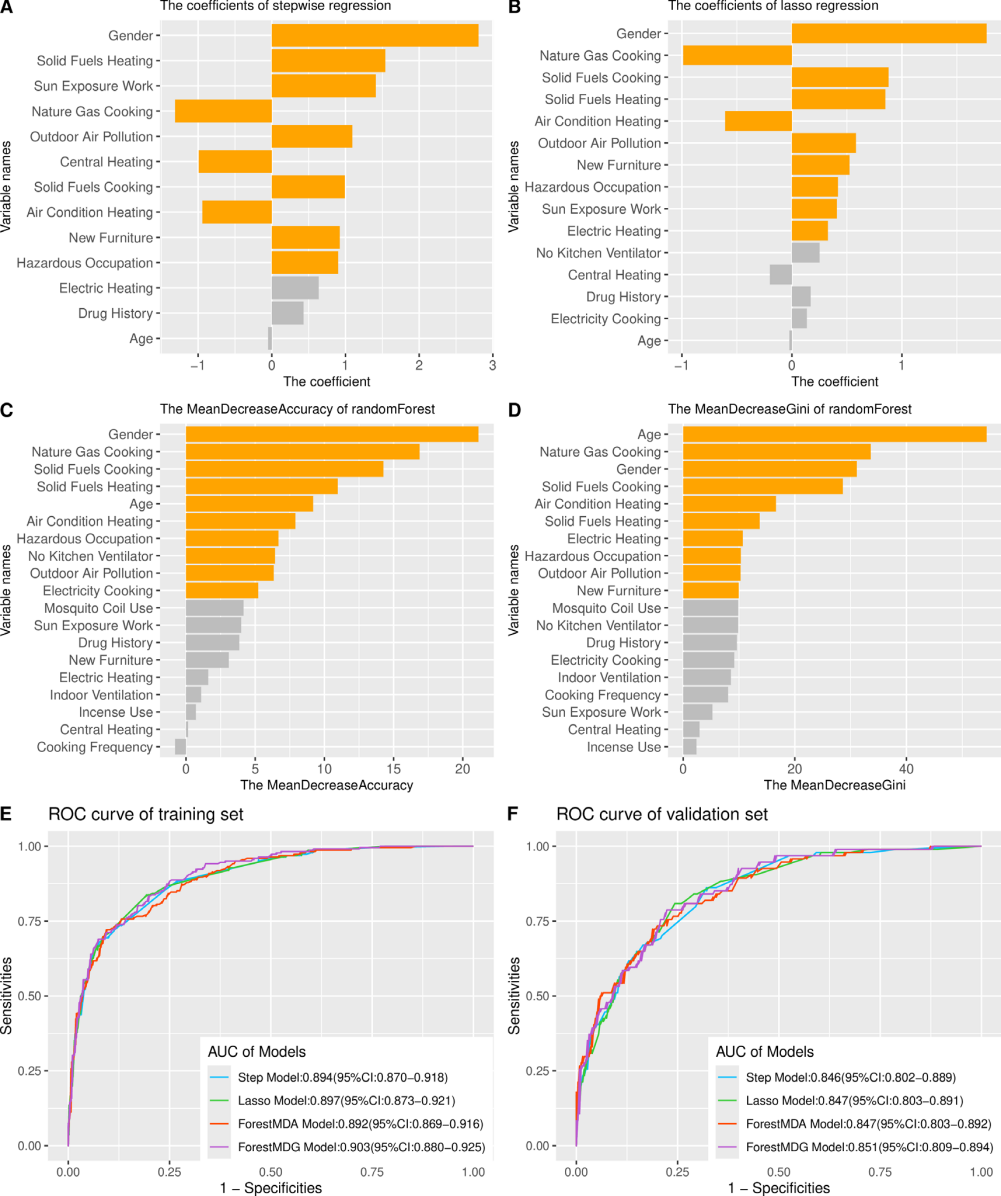
Feature importance ranking of the four models and ROC curves of the models for predicting SLE onset relative risk. (**A**) Coefficient ranking of stepwise regression. (**B**) Coefficient ranking of lasso regression. (**C**) The MeanDecreaseAccuracy of random forest. (**D**) The MeanDecreaseGini of random forest. (**E**) ROC curves for the training set and (**F**) ROC curves for the validation set. AUC, area under the ROC curve; ROC, receiver operating characteristic.

We selected age, gender and factors significantly associated with SLE occurrence after adjusting for age and gender. In the training set, these variables were analysed using forward–backward multivariate binary logistic regression, Lasso regression and random forest models (online supplemental figure S1). Coefficients and importance scores for each variable were obtained from these models (figure 3A–D). Among these models, we selected the top 10 variables for binary logistic regression to form four models.

The Step model comprises 10 features: gender, solid fuels cooking, sun exposure work, nature gas cooking, outdoor air pollution, central heating, solid fuels heating, air condition heating, new furniture, hazardous occupation. The Lasso model encompasses 10 features: gender, nature gas cooking, solid fuels cooking, solid fuels heating, air condition heating, outdoor air pollution, new furniture, hazardous occupation, sun exposure work, electric heating. The ForestMDA model includes 10 features: gender, nature gas cooking, solid fuels cooking, solid fuels heating, age, air condition heating, hazardous occupation, no kitchen ventilator, outdoor air pollution, electricity cooking. The ForestMDG model consists of 10 features: age, nature gas cooking, gender, solid fuels cooking, solid fuels heating, electric heating, hazardous occupation, outdoor air pollution, new furniture.

### Model performance evaluation and cross-validation

The discriminative ability of the models was evaluated using AUC values. During the training phase (figure 3E), the AUC values for the models were as follows: Step model 0.894 (95% CI 0.870 to 0.918), Lasso model 0.897 (95% CI 0.873 to 0.921), ForestMDA model 0.892 (95% CI 0.869 to 0.916) and ForestMDG model 0.903 (95% CI 0.880 to 0.925). In the validation set (figure 3F), the AUC values slightly decreased as follows: Step model 0.846 (95% CI 0.802 to 0.889), Lasso model 0.847 (95% CI 0.803 to 0.891), ForestMDA model 0.847 (95% CI 0.803 to 0.892) and ForestMDG model 0.851 (95% CI 0.809 to 0.894).

**Figure 4:**
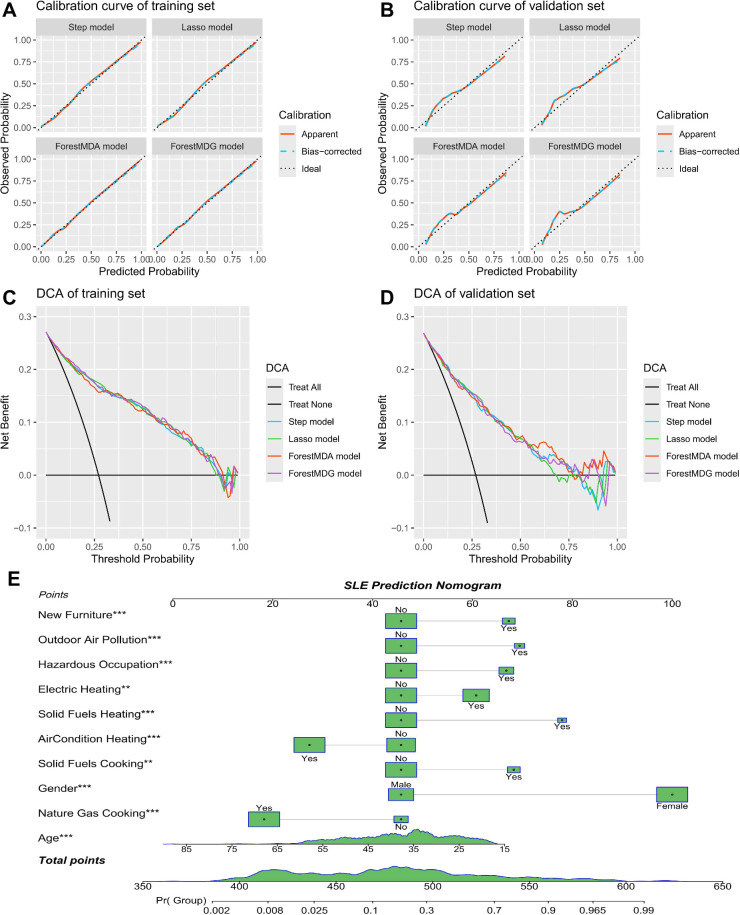
Calibration plots, decision curve analysis and dynamic nomogram for predicting the relative risk of SLE onset. Calibration plots of the models in the training set (**A**) and the validation set (**B**). Decision curve analysis for the training set (**C**) and the validation set (**D**). The dynamic nomogram (**E**). Electric heating, such as electric grills or radiant heaters; points for each predictor were determined by marking a marker on the ‘Points’ axis, and the cumulative score was computed as the sum of these points. The likelihood of developing SLE can be easily derived from the total score. Significance levels: *p<0.05, **p<0.01, ***p<0.001. DCA, decision curve analysis; SLE, systemic lupus erythematosus.

The Hosmer-Lemeshow test indicated an adequate fit for all four models (p>0.05). Across the training and validation cohorts, calibration curves demonstrated that the predicted and actual probabilities of SLE onset for each model were closely aligned (figure 4A,B). DCA curves showed that each model had clinical utility within a certain probability threshold across the training and validation cohorts (figure 4C,D).

We also computed the net reclassification index (NRI) and integrated discrimination improvement index (IDI). The ForestMDG model showed an NRI of 0.2337 (95% CI 0.1057 to 0.3617) compared with the Step model, with a non-significant IDI. Relative to the Lasso model, the ForestMDG model had an NRI of 0.1531 (95% CI 0.025 to 0.2811), also with a non-significant IDI. Compared with the ForestMDA model, the ForestMDG model had an NRI of 0.3302 (95% CI 0.2025 to 0.4579) and an IDI of 0.0143 (95% CI 0.0027 to 0.0259). Finally, leave-one-out cross-validation was performed, yielding the best performance for the ForestMDG model with an accuracy of 0.8338 (online supplemental figure S2A).

### Nomogram for early prediction of SLE relative risk

Prediction of the overall data using the ForestMDG model yielded an optimal critical value of 0.247, with sensitivity measured at 81.96% and specificity at 78.97%. Employing the specified threshold, our study predicted 438 patients with SLE and 729 patients with non-SLE (online supplemental figure S2B). Based on these independent environmental factors, we developed a predictive nomogram (figure 4E). Each factor in the nomogram corresponds to a specific point on the ‘point’ scale. A perpendicular line is drawn for every factor reaching the ‘point’ axis to determine its score. The scores are added together to produce a total score, which is then applied to the ‘risk’ axis to estimate the probability of SLE occurrence. A greater overall score corresponds to an increased risk of SLE, whereas a lower score signifies a reduced risk.

Additionally, we created a dynamic nomogram with an intuitive web interface for practical use and to view the specific model parameters. (online supplemental figure S3). A dynamic nomogram is accessible via the following link: https://yingzhang99321.shinyapps.io/dynnomapp/.

## Discussion

In this case–control study of SLE, we investigated basic demographic information, occupational exposures and living environmental exposures to develop a more accurate prediction model for SLE relative risk using three feature screening methods, ultimately utilising Random Forest for feature selection. The leave-one-out cross-validation method demonstrated an accuracy of 0.8338 for risk assessment based on easily collected living environment characteristics. Our findings indicated that heating methods, cooking methods, occupational exposure to hazardous substances, outdoor air pollution and organic pollutants emitted from indoor furniture strongly influence the development of SLE. These findings emphasise the crucial role that environmental influences have in the development of SLE, underscoring the necessity for increased focus on these aspects in prevention and management strategies.

In our ForestMDG model, female gender is identified as the most significant risk factor for SLE, consistent with current research. A study utilising a Bayesian hierarchical linear mixed model estimated that the global incidence of SLE among women is five times higher than among men, underscoring the importance of gender in SLE occurrence.^6 24^ Mechanistic studies suggest that the higher risk in women could be attributed to the influence of oestrogen, improper X chromosome silencing, elevated production of toll-like receptor gene products and modifications in microRNA activity.^3 25^ Our study of patients with SLE aged 18 and above reveals a higher risk of SLE in women of childbearing age, with a gradual decrease after menopause, aligning with mainstream research findings.^25^,^27^

Regarding occupational exposure to hazardous substances, we found a 2.24-fold increase in SLE risk among individuals with such exposure, even after adjusting for age and gender. Related studies indicate that exposure to pesticides, chemicals and industrial pollutants can increase SLE risk.^28^ One study determined that exposure to pesticides increased the risk of SLE by 2.24 times compared with those not exposed (OR 2.24, 95% CI 1.28 to 3.93).^29^ Mechanistic research suggests that prolonged high-level pesticide exposure may contribute to SLE development or exacerbation by significantly affecting T lymphocyte subsets and cytokine expression, causing DNA damage and oxidative stress.^30 31^

There is a significant relationship between cooking and heating methods and the risk of SLE. Using natural gas for cooking and air conditioners for heating are protective factors, while using conventional solid fuels for cooking and heating, as well as electrical appliances (other than air conditioners) for heating, increases the risk of SLE. Natural gas, as a clean fuel,^32^ produces only carbon dioxide and water during combustion, posing no harm to humans. Air conditioning provides warmth without indoor pollution. However, burning conventional solid fuels like coal and wood emits pollutants such as carbon oxides, volatile organic compounds (VOCs), nitrogen oxides, PM and PAHs.^33 34^ Studies show that PAH concentrations in kitchens using solid fuels exceed outdoor air levels and surpass Chinese health protection standards.^35 36^ Aside from air conditioning, other electric heating methods may increase SLE risk. Infrared heaters generate localised high temperatures and intense light, causing skin inflammation and erythema.^37^ High temperatures can also release harmful substances like bisphenol A and phthalates from surrounding plastic materials, increasing SLE risk in susceptible individuals.^38^

Poor outdoor air quality is linked to a 3.2-fold higher risk of SLE after adjusting for age and gender. Proximity to smoky factories likely impacts air quality, producing PM and environmental pollutants detrimental to health. Studies show higher autoimmune disease likelihood with elevated PM2.5 levels,^39^ and long-term air pollution exacerbates SLE symptoms in lupus-prone mice.^40^ New indoor furniture is a significant risk factor for SLE, likely due to the release of indoor air pollutants. Smoking, solvent use, renovations and household products are sources of VOCs, with formaldehyde levels higher in new houses and furniture.^41^ VOCs significantly impact human health, with exposure linked to inflammatory and autoimmune diseases. VOC exposure leads to the generation of reactive oxygen species, resulting in oxidative stress and tissue damage. The resulting inflammatory response triggers immune cells to produce cytokines and chemokines, potentially leading to autoimmune diseases. Excess VOCs may also promote autoimmunity by altering DNA methylation in CD4 T cells.^42 43^

Our research revealed a strong connection between environmental factors and the risk of SLE, demonstrating that identifying high-risk groups through living environment variables is both simple and effective. Clinicians can use our model to predict SLE relative risk more accurately and earlier. This can guide high-risk individuals, especially those with a family history of SLE, to adjust their lifestyles and modify their work or living environments, aiming for primary prevention and early intervention to prevent this debilitating disease.

Our study has several limitations: (1) the study adopted a case–control design to collect past environmental exposure factors, which may be susceptible to recall bias. (2) Our study has inherent selection bias, as the patients were exclusively recruited from Central South University’s Xiangya Second Hospital, which may limit the representativeness of the sample. Furthermore, the healthy controls were drawn from a health check-up centre, potentially introducing bias, as healthier or more health-conscious individuals may have been more likely to participate. These factors may affect the generalisability of our findings. (3) Environmental exposure factors of participants were collected through questionnaires, with most features being qualitative data, and there was limited quantitative data for comprehensive prediction. (4) The study lacked external validation datasets, although internal cross-validation was conducted, further validation of the results is warranted. (5) Since the occurrence of SLE is attributed to the interaction between genetic and environmental factors, our study did not address genetic factors.

### Conclusion

To summarise, we created a user-friendly dynamic nomogram based on environmental exposures to predict the relative risk of SLE occurrence compared with individuals not reporting similar environmental risk factors. This tool can assist in evaluating the relative risk of SLE under daily environmental exposures and can guide high-risk individuals in changing their health habits and modifying their work or living environments, thereby serving as primary prevention to reduce the risk of SLE occurrence.

## supplementary material

10.1136/lupus-2024-001311 online supplemental file 1

## Data Availability

Data are available upon reasonable request.
