# Supplementary material for: Development of a predictive model for systemic lupus erythematosus incidence risk based on environmental exposure factors
Source: Lupus Sci Med. 2024 Nov 20;11(2):e001311. doi: 10.1136/lupus-2024-001311 (PMC11580284; doi:10.1136/lupus-2024-001311)
Supplement: online supplemental file 1 [file lupus-11-2-s001.pdf]

Development of a Predictive Model for Systemic Lupus Erythematosus Incidence Risk Based on Environmental Exposure Factor

Contents

Supplementary Table S1. Variable Name and Assignment Comments. 2
Supplementary Table S2. Comparison of baseline features between SLE and control groups in training and validation sets. 5
Supplementary Table S3. Comparison of baseline features between SLE and control groups in training and validation sets. 7
Supplementary Table S4. Univariate and multivariate logistic regression analyses of risk factors associated with the development of SLE 10
Supplementary Figure S1. Random forest and Lasso regression analysis 12
Supplementary Figure S2. Accuracy of different Models and Prediction of the data set by the best model. 12
Supplementary Figure S3. Online dynamic nomogram for predicting the risk of SLE onset. 13

Supplementary Table S1. Variable Name and Assignment Comments.

Variable name	Variable labels	Assignment	The meaning of an assignment
Age	Age	-	Age
Gender	Gender	Male	Male
		Female	Female
Cigarette smoking	Current smoking status	No	Have quit smoking for more than 1 month or have never smoked
		Yes	Cigarette smoking
Passive smoking	Passive Smoke	No	Basically no passive smoking
		<= 1 hour / day	Have passive smoking, not more than 1 hour per day on average
		> 1 hour / day	Have passive smoking, averaging more than 1 hour per day
History of drug use within 1 year	Drug History	No	No
		Yes	Yes
Hazardous Substances Exposure Occupation	Hazardous Occupation	No	No
		Yes	Yes (worked in at least one of the following occupations: coke oven worker,firefighter, car driver, traffic policeman, road toll collector, cook, miner in underground operations, construction worker, glass maker, quarryman/rock crusher, electronics/chip maker,ceramics maker, mercury thermometer/sphygmomanometer maker, printer/copyist, electronics/chip maker,battery maker, mechanic, painter, oil/gas processor, plastics maker, cosmetic maker, maker of cleaning products such as soap/laundry detergent/hand sanitizer, artisan with regular contact with paints, hairdresser, manicurist, drug maker, pesticide or insecticide maker/sprayer, jobs with potential exposure to radiation such as nuclear power plants/radiation workers, research/technology workers with potential exposure to toxic substances, other jobs with potential exposure to toxic substances or health effects)

Sunshine High Exposure Occupation	Sun Exposure Occupation	No	No
		Yes	Yes
Have been exposed to large odors Furniture or objects	New Furniture	No	No
		Yes	Yes
Outdoor air pollution at residence or workplace	Outdoor Air Pollution	Good	No
		Wrong	Defined when one of the following is met：<br>Live or work near industries that emit smoke from chimneys;<br>Other conditions that may affect air quality
Indoor ventilation at residence or workplace	Indoor Ventilation	Good	Good (two-way ventilation, good air circulation)
		Wrong	Fair (windows but poor air circulation) or Poor (no windows)
Lack of exhaust devices in kitchens	No Kitchen Ventilator	No	No
		Yes	Yes
Frequency of cooking	Cooking Frequency	<= 2 days/week	Cooking 1-2 days a week
		> 2 days /week	Cooking more than 2 days a week
Cooking with traditional solid fuels	Solid Fuels Cooking	No	No
		Yes	Defined when one of the following is met：<br>Burning coal/charcoal for cooking；<br>Wood burning for cooking；<br>Cooking with other fuels
Burning natural gas for cooking	Nature Gas Cooking	No	No
		Yes	Yes
Cook on electric power	Electricity Cooking	No	No
		Yes	Yes
Traditional solid fuel heating	Solid Fuels Heating	No	No

		Yes	Defined when one of the following is met：<br>Burning coal stoves for heating;<br>Make a fire bed to keep warm;<br>Burning wall heating;<br>Burn a fire pit to keep warm;<br>Other fuel heating.
Air conditioning and heating	Air Condition Heating	No	No
		Yes	Yes
Central Heating	Central Heating	No	No
		Yes	Yes
Electric heating	Electric Heating	No	No
		Yes	Yes (Electric heating wire luminous heat baking fire, etc.)
Frequency of mosquito coil burning	Mosquito Coil Use	No	No
		Yes	Yest
Frequency of burning incense	Incense Use	Infrequent	Infrequent
		Frequent	Frequent

Supplementary Table S2. Comparison of baseline features in training and validation sets.

Data	Training Set (N=818)	Validation Set (N=349)	Total (N=1167)	p
Group				1
Control	596 (72.86%)	255 (73.07%)	851 (72.92%)	
SLE	222 (27.14%)	94 (26.93%)	316 (27.08%)	
Age	36.00 [30.00;45.00]	36.00 [30.00;46.00]	36.00 [30.00;45.00]	0.957
Gender				0.271
Male	325 (39.73%)	126 (36.10%)	451 (38.65%)	
Female	493 (60.27%)	223 (63.90%)	716 (61.35%)	
Current Smoking Status				0.307
No	672 (82.15%)	296 (84.81%)	968 (82.95%)	
Yes	146 (17.85%)	53 (15.19%)	199 (17.05%)	
Passive Smoke				0.337
No	428 (52.32%)	171 (49.00%)	599 (51.33%)	
<= 1 hour/day	290 (35.45%)	125 (35.82%)	415 (35.56%)	
> 1 hour/day	100 (12.22%)	53 (15.19%)	153 (13.11%)	
Drug History				0.873
No	624 (76.28%)	264 (75.64%)	888 (76.09%)	
Yes	194 (23.72%)	85 (24.36%)	279 (23.91%)	
Hazardous Occupation				0.578
No	667 (81.54%)	279 (79.94%)	946 (81.06%)	
Yes	151 (18.46%)	70 (20.06%)	221 (18.94%)	
Sun Exposure Work				0.003
No	764 (93.40%)	307 (87.97%)	1071 (91.77%)	
Yes	54 (6.60%)	42 (12.03%)	96 (8.23%)	
Outdoor Air Pollution				0.78
No	737 (90.10%)	317 (90.83%)	1054 (90.32%)	
Yes	81 (9.90%)	32 (9.17%)	113 (9.68%)	
Indoor Ventilation				0.593
Good	543 (66.38%)	238 (68.19%)	781 (66.92%)	
Wrong	275 (33.62%)	111 (31.81%)	386 (33.08%)	
New Furniture				0.735
No	700 (85.57%)	302 (86.53%)	1002 (85.86%)	
Yes	118 (14.43%)	47 (13.47%)	165 (14.14%)	
No Kitchen Ventilator				0.695
No	730 (89.24%)	308 (88.25%)	1038 (88.95%)	
Yes	88 (10.76%)	41 (11.75%)	129 (11.05%)	
Cooking Frequency				0.419
<= 2 days/week	572 (69.93%)	235 (67.34%)	807 (69.15%)	
> 2 days/week	246 (30.07%)	114 (32.66%)	360 (30.85%)	
Solid Fuels Cooking				0.423
No	701 (85.70%)	292 (83.67%)	993 (85.09%)	
Yes	117 (14.30%)	57 (16.33%)	174 (14.91%)	

Nature Gas Cooking				0.405
No	141 (17.24%)	68 (19.48%)	209 (17.91%)	
Yes	677 (82.76%)	281 (80.52%)	958 (82.09%)	
Electricity Cooking				0.881
No	675 (82.52%)	286 (81.95%)	961 (82.35%)	
Yes	143 (17.48%)	63 (18.05%)	206 (17.65%)	
Solid Fuels Heating				0.965
No	757 (92.54%)	322 (92.26%)	1079 (92.46%)	
Yes	61 (7.46%)	27 (7.74%)	88 (7.54%)	
Air Condition Heating				0.198
No	375 (45.84%)	145 (41.55%)	520 (44.56%)	
Yes	443 (54.16%)	204 (58.45%)	647 (55.44%)	
Central Heating				1
No	750 (91.69%)	320 (91.69%)	1070 (91.69%)	
Yes	68 (8.31%)	29 (8.31%)	97 (8.31%)	
Electric Heating				1
No	475 (58.07%)	202 (57.88%)	677 (58.01%)	
Yes	343 (41.93%)	147 (42.12%)	490 (41.99%)	
Mosquito Coil Use				0.721
No	329 (40.22%)	145 (41.55%)	474 (40.62%)	
Yes	489 (59.78%)	204 (58.45%)	693 (59.38%)	
Incense Use				0.552
Infrequent	795 (97.19%)	342 (97.99%)	1137 (97.43%)	
Frequent	23 (2.81%)	7 (2.01%)	30 (2.57%)	

Supplementary Table S3. Comparison of baseline features between SLE and control groups in training and validation sets.

Data	Training Set				Validation Set			
Group	Control (N=596)	SLE (N=222)	Total (N=818)	p	Control (N=255)	SLE (N=94)	Total (N=349)	p
Age	38.00 [31.00;46.00]	34.00 [25.00;42.00]	36.00 [30.00;45.00]	< 0.001	37.00 [31.00;48.00]	33.00 [27.00;42.00]	36.00 [30.00;46.00]	0.009
Gender				< 0.001				< 0.001
Male	308 (51.68%)	17 (7.66%)	325 (39.73%)		120 (47.06%)	6 (6.38%)	126 (36.10%)	
Female	288 (48.32%)	205 (92.34%)	493 (60.27%)		135 (52.94%)	88 (93.62%)	223 (63.90%)	
Current Smoking Status				< 0.001				0.003
No	464 (77.85%)	208 (93.69%)	672 (82.15%)		207 (81.18%)	89 (94.68%)	296 (84.81%)	
Yes	132 (22.15%)	14 (6.31%)	146 (17.85%)		48 (18.82%)	5 (5.32%)	53 (15.19%)	
Passive Smoke				0.012				0.585
No	293 (49.16%)	135 (60.81%)	428 (52.32%)		121 (47.45%)	50 (53.19%)	171 (49.00%)	
<= 1 hour/day	225 (37.75%)	65 (29.28%)	290 (35.45%)		93 (36.47%)	32 (34.04%)	125 (35.82%)	
> 1 hour/day	78 (13.09%)	22 (9.91%)	100 (12.22%)		41 (16.08%)	12 (12.77%)	53 (15.19%)	
Drug History				< 0.001				0.464
No	476 (79.87%)	148 (66.67%)	624 (76.28%)		196 (76.86%)	68 (72.34%)	264 (75.64%)	
Yes	120 (20.13%)	74 (33.33%)	194 (23.72%)		59 (23.14%)	26 (27.66%)	85 (24.36%)	
Hazardous Occupation				0.003				0.161
No	501 (84.06%)	166 (74.77%)	667 (81.54%)		209 (81.96%)	70 (74.47%)	279 (79.94%)	
Yes	95 (15.94%)	56 (25.23%)	151 (18.46%)		46 (18.04%)	24 (25.53%)	70 (20.06%)	
Sun Exposure Work				0.125				0.417
No	562 (94.30%)	202 (90.99%)	764 (93.40%)		227 (89.02%)	80 (85.11%)	307 (87.97%)	
Yes	34 (5.70%)	20 (9.01%)	54 (6.60%)		28 (10.98%)	14 (14.89%)	42 (12.03%)	
Outdoor Air Pollution				< 0.001				0.001
Good	561 (94.13%)	176 (79.28%)	737 (90.10%)		240 (94.12%)	77 (81.91%)	317 (90.83%)	

Wrong	35 (5.87%)	46 (20.72%)	81 (9.90%)		15 (5.88%)	17 (18.09%)	32 (9.17%)	
Indoor Ventilation				0.003				0.006
Good	414 (69.46%)	129 (58.11%)	543 (66.38%)		185 (72.55%)	53 (56.38%)	238 (68.19%)	
Wrong	182 (30.54%)	93 (41.89%)	275 (33.62%)		70 (27.45%)	41 (43.62%)	111 (31.81%)	
New Furniture				< 0.001				0.175
No	529 (88.76%)	171 (77.03%)	700 (85.57%)		225 (88.24%)	77 (81.91%)	302 (86.53%)	
Yes	67 (11.24%)	51 (22.97%)	118 (14.43%)		30 (11.76%)	17 (18.09%)	47 (13.47%)	
No Kitchen Ventilator				< 0.001				< 0.001
No	566 (94.97%)	164 (73.87%)	730 (89.24%)		246 (96.47%)	62 (65.96%)	308 (88.25%)	
Yes	30 (5.03%)	58 (26.13%)	88 (10.76%)		9 (3.53%)	32 (34.04%)	41 (11.75%)	
Cooking Frequency				0.248				0.002
<= 2 days/week	424 (71.14%)	148 (66.67%)	572 (69.93%)		184 (72.16%)	51 (54.26%)	235 (67.34%)	
> 2 days/week	172 (28.86%)	74 (33.33%)	246 (30.07%)		71 (27.84%)	43 (45.74%)	114 (32.66%)	
Solid Fuels Cooking				< 0.001				< 0.001
No	568 (95.30%)	133 (59.91%)	701 (85.70%)		234 (91.76%)	58 (61.70%)	292 (83.67%)	
Yes	28 (4.70%)	89 (40.09%)	117 (14.30%)		21 (8.24%)	36 (38.30%)	57 (16.33%)	
Nature Gas Cooking				< 0.001				< 0.001
No	39 (6.54%)	102 (45.95%)	141 (17.24%)		32 (12.55%)	36 (38.30%)	68 (19.48%)	
Yes	557 (93.46%)	120 (54.05%)	677 (82.76%)		223 (87.45%)	58 (61.70%)	281 (80.52%)	
Electricity Cooking				< 0.001				0.155
No	520 (87.25%)	155 (69.82%)	675 (82.52%)		214 (83.92%)	72 (76.60%)	286 (81.95%)	
Yes	76 (12.75%)	67 (30.18%)	143 (17.48%)		41 (16.08%)	22 (23.40%)	63 (18.05%)	
Solid Fuels Heating				< 0.001				< 0.001
No	580 (97.32%)	177 (79.73%)	757 (92.54%)		249 (97.65%)	73 (77.66%)	322 (92.26%)	
Yes	16 (2.68%)	45 (20.27%)	61 (7.46%)		6 (2.35%)	21 (22.34%)	27 (7.74%)	
AirCondition Heating				< 0.001				< 0.001

No	219 (36.74%)	156 (70.27%)	375 (45.84%)		83 (32.55%)	62 (65.96%)	145 (41.55%)	
Yes	377 (63.26%)	66 (29.73%)	443 (54.16%)		172 (67.45%)	32 (34.04%)	204 (58.45%)	
Central Heating				0.001				0.02
No	534 (89.60%)	216 (97.30%)	750 (91.69%)		228 (89.41%)	92 (97.87%)	320 (91.69%)	
Yes	62 (10.40%)	6 (2.70%)	68 (8.31%)		27 (10.59%)	2 (2.13%)	29 (8.31%)	
Electric Heating				< 0.001				0.03
No	378 (63.42%)	97 (43.69%)	475 (58.07%)		157 (61.57%)	45 (47.87%)	202 (57.88%)	
Yes	218 (36.58%)	125 (56.31%)	343 (41.93%)		98 (38.43%)	49 (52.13%)	147 (42.12%)	
Mosquito Coil Use				0.002				0.109
No	260 (43.62%)	69 (31.08%)	329 (40.22%)		113 (44.31%)	32 (34.04%)	145 (41.55%)	
Yes	336 (56.38%)	153 (68.92%)	489 (59.78%)		142 (55.69%)	62 (65.96%)	204 (58.45%)	
Incense Use				0.121				0.088
Infrequent	583 (97.82%)	212 (95.50%)	795 (97.19%)		252 (98.82%)	90 (95.74%)	342 (97.99%)	
Frequent	13 (2.18%)	10 (4.50%)	23 (2.81%)		3 (1.18%)	4 (4.26%)	7 (2.01%)	

.

Supplementary Table S4. Univariate and multivariate logistic regression analyses of risk factors associated with the development of SLE

name	desc	Control (N=851)	SLE (N=316)	OR(95%CI,Pvalue)	adjOR(95%CI,Pvalue)
Age	Mean ± SD	38.8 ± 11.0	35.1 ± 10.8	0.97(0.96-0.98, p<.001)	
Gender	Male	428 (50.3%)	23 (7.3%)		
	Female	423 (49.7%)	293 (92.7%)	12.89(8.26-20.12, p<.001)	
Current Smoking Status	No	671 (78.8%)	297 (94%)		
	Yes	180 (21.2%)	19 (6%)	0.24(0.15-0.39, p<.001)	1.15(0.62-2.12, p=.658)
Passive Smoke	No	414 (48.6%)	185 (58.5%)		
	<= 1hour/day	318 (37.4%)	97 (30.7%)	0.68(0.51-0.91, p=.009)	0.76(0.56-1.04, p=.087)
	> 1hour/day	119 (14%)	34 (10.8%)	0.64(0.42-0.97, p=.036)	0.96(0.6-1.54, p=.861)
Drug History	No	672 (79%)	216 (68.4%)		
	Yes	179 (21%)	100 (31.6%)	1.74(1.30-2.32, p<.001)	1.9(1.37-2.63, p<.001)
Hazardous Occupation	No	710 (83.4%)	236 (74.7%)		
	Yes	141 (16.6%)	80 (25.3%)	1.71(1.25-2.33, p<.001)	3.24(2.19-4.79, p<.001)
Sun Exposure Work	No	789 (92.7%)	282 (89.2%)		
	Yes	62 (7.3%)	34 (10.8%)	1.53(0.99-2.38, p=.056)	4.08(2.33-7.14, p<.001)
Outdoor Air Pollution	No	801 (94.1%)	253 (80.1%)		
	Yes	50 (5.9%)	63 (19.9%)	3.99(2.68-5.93, p<.001)	4.2(2.65-6.66, p<.001)
Indoor Ventilation	Good	599 (70.4%)	182 (57.6%)		
	Wrong	252 (29.6%)	134 (42.4%)	1.75(1.34-2.29, p<.001)	1.51(1.13-2.03, p=.006)
New Furniture	No	754 (88.6%)	248 (78.5%)		
	Yes	97 (11.4%)	68 (21.5%)	2.13(1.51-3.00, p<.001)	1.98(1.36-2.9, p<.001)
No Kitchen Ventilator	No	812 (95.4%)	226 (71.5%)		
	Yes	39 (4.6%)	90 (28.5%)	8.29(5.54-12.41, p<.001)	6.17(3.96-9.63, p<.001)
Cooking Frequency	<=2 times/week	608 (71.4%)	199 (63%)		
	>2 times/week	243 (28.6%)	117 (37%)	1.47(1.12-1.93, p=.005)	1.68(1.21-2.32, p=.002)

Solid Fuels Cooking	No	802 (94.2%)	191 (60.4%)		
	Yes	49 (5.8%)	125 (39.6%)	10.71(7.43-15.45, p<.001)	9.43(6.26-14.21, p<.001)
Nature Gas Cooking	No	71 (8.3%)	138 (43.7%)		
	Yes	780 (91.7%)	178 (56.3%)	0.12(0.08-0.16, p<.001)	0.15(0.1-0.21, p<.001)
Electricity Cooking	No	734 (86.3%)	227 (71.8%)		
	Yes	117 (13.7%)	89 (28.2%)	2.46(1.80-3.36, p<.001)	2.14(1.5-3.04, p<.001)
Solid Fuels Heating	No	829 (97.4%)	250 (79.1%)		
	Yes	22 (2.6%)	66 (20.9%)	9.95(6.02-16.45, p<.001)	9.58(5.44-16.86, p<.001)
AirCondition Heating	No	302 (35.5%)	218 (69%)		
	Yes	549 (64.5%)	98 (31%)	0.25(0.19-0.33, p<.001)	0.23(0.17-0.31, p<.001)
Central Heating	No	762 (89.5%)	308 (97.5%)		
	Yes	89 (10.5%)	8 (2.5%)	0.22(0.11-0.46, p<.001)	0.23(0.11-0.48, p<.001)
Electric Heating	No	535 (62.9%)	142 (44.9%)		
	Yes	316 (37.1%)	174 (55.1%)	2.07(1.60-2.70, p<.001)	1.85(1.39-2.47, p<.001)
Mosquito Coil Use	No	373 (43.8%)	101 (32%)		
	Yes	478 (56.2%)	215 (68%)	1.66(1.26-2.18, p<.001)	1.84(1.37-2.48, p<.001)
Incense Use	Infrequent	835 (98.1%)	302 (95.6%)		
	Frequent	16 (1.9%)	14 (4.4%)	2.42(1.17-5.02, p=.018)	2.43(1.06-5.56, p=.036)

OR, odds ratio; CI, confidence interval; adjOR, adjust odds ratio; ORs were adjusted for gender and age.

Supplementary Figure S1. Random forest and Lasso regression analysis

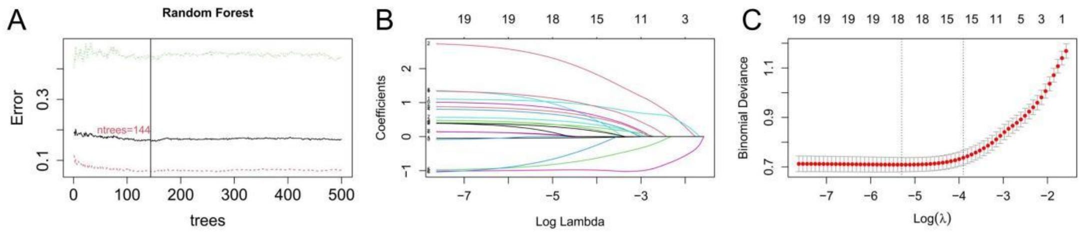

(A) Variation of error rate with the number of decision trees. (B) Path diagram of Lasso regression coefficients for 27 factors. (C) Cross-validation curves.

Supplementary Figure S2. Accuracy of different Models and Prediction of the data set by the best model.

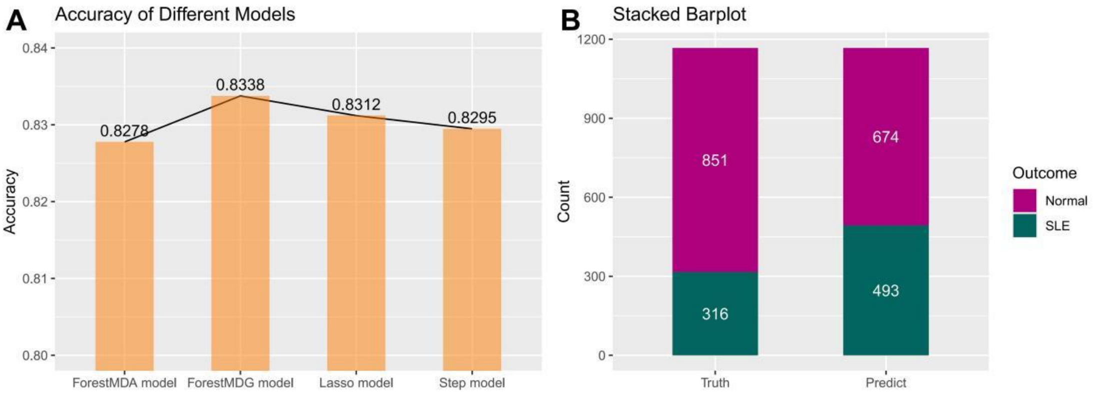

(A) Accuracy of leave-one-out cross-validated models. (B) Prediction of the data set by the best cutoff value of the ForestMDG model.

Supplementary Figure S3. Online dynamic nomogram for predicting the relative risk of SLE onset.

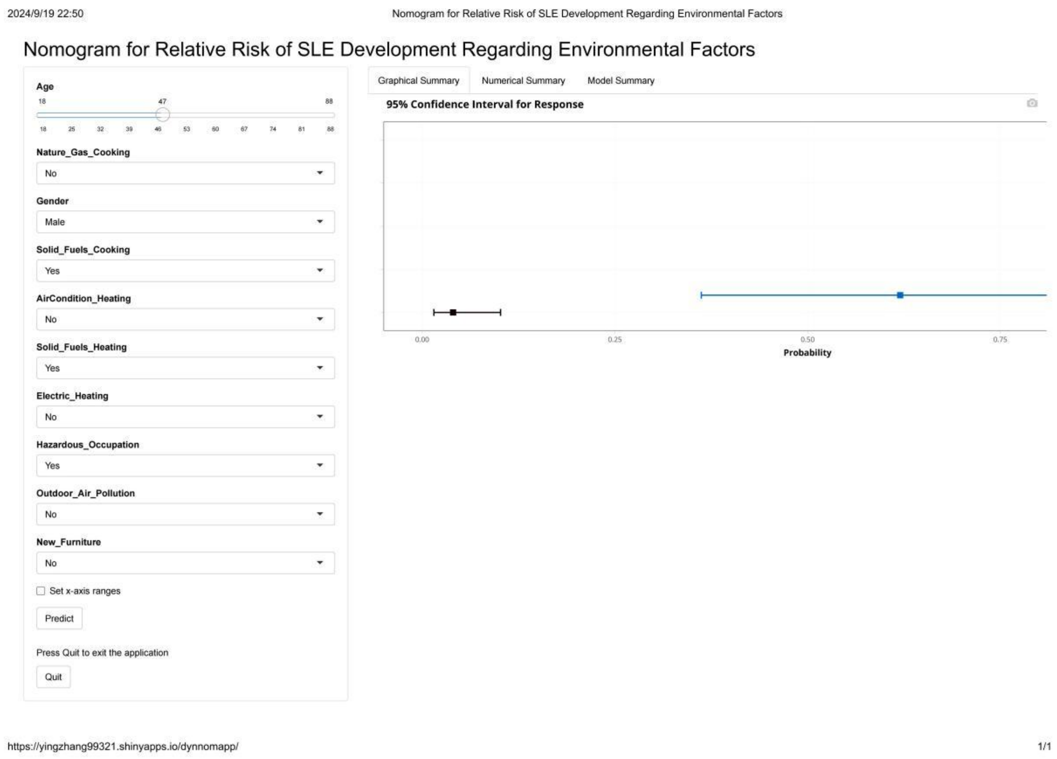

The intuitive interface of the online dynamic nomogram (https://yingzhang99321.shinyapps.io/dynnomapp/). When no risk factors and protective factors are present, the risk of SLE occurrence is 0. Upon selecting relevant conditions and clicking the "predict" button, the tool displays the relative risk of SLE along with its 95% confidence interval.
